# REST–Mediated Recruitment of Polycomb Repressor Complexes in Mammalian Cells

**DOI:** 10.1371/journal.pgen.1002494

**Published:** 2012-03-01

**Authors:** Nikolaj Dietrich, Mads Lerdrup, Eskild Landt, Shuchi Agrawal-Singh, Mads Bak, Niels Tommerup, Juri Rappsilber, Erik Södersten, Klaus Hansen

**Affiliations:** 1 Biotech Research and Innovation Centre (BRIC) and Centre for Epigenetics, University of Copenhagen, Copenhagen, Denmark; 2 Wilhelm Johannsen Centre For Functional Genome Research, Department of Cellular and Molecular Medicine, University of Copenhagen, Copenhagen, Denmark; 3 Wellcome Trust Centre for Cell Biology, University of Edinburgh, Edinburgh, United Kingdom; University of California San Francisco, United States of America

## Abstract

Polycomb Repressive Complex (PRC) 1 and PRC2 regulate genes involved in differentiation and development. However, the mechanism for how PRC1 and PRC2 are recruited to genes in mammalian cells is unclear. Here we present evidence for an interaction between the transcription factor REST, PRC1, and PRC2 and show that RNF2 and REST co-regulate a number of neuronal genes in human teratocarcinoma cells (NT2-D1). Using NT2-D1 cells as a model of neuronal differentiation, we furthermore showed that retinoic-acid stimulation led to displacement of PRC1 at REST binding sites, reduced H3K27Me3, and increased gene expression. Genome-wide analysis of Polycomb binding in *Rest−/−* and *Eed−/−* mouse embryonic stem (mES) cells showed that Rest was required for PRC1 recruitment to a subset of Polycomb regulated neuronal genes. Furthermore, we found that PRC1 can be recruited to Rest binding sites independently of CpG islands and the H3K27Me3 mark. Surprisingly, PRC2 was frequently increased around Rest binding sites located in CpG-rich regions in the *Rest−/−* mES cells, indicating a more complex interplay where Rest also can limit PRC2 recruitment. Therefore, we propose that Rest has context-dependent functions for PRC1- and PRC2- recruitment, which allows this transcription factor to act both as a recruiter of Polycomb as well as a limiting factor for PRC2 recruitment at CpG islands.

## Author Summary

Multicellular organisms are composed of a large number of specialized cell types that all originate from the Embryonic Stem cell (ES cell). It is crucial for the maintenance of naïve ES cells that developmental genes are kept in an off-state until appropriate differentiation stimuli are received. Polycomb Repressive Complexes, PRC1 and PRC2, are bound at and repress the activity of a large number of key developmental genes in ES cells and at different stages of differentiation. While in *Drosophila* the PRC complexes are recruited to DNA elements called Polycomb Response Elements (PREs), through the interaction with transcription factors; examples of such factors remain poorly characterized in mammals. We here demonstrate that the transcription factor Rest interacts with and is required for recruitment of PRC1 and PRC2 to a subset of Rest target genes in mouse embryonic stem (mES) cells. In line with REST being a repressor of neuronal genes, we found that PRC1 and PRC2 co-localized with REST at genes involved in neuronal development and got displaced during neuronal differentiation. Based on our data we propose that the PRC1 and PRC2 complexes function as co-repressors for Rest to control the timed expression of developmental genes in the process of cellular differentiation.

## Introduction

Polycomb group (PcG) proteins are epigenetic regulators of gene expression and play an essential role during embryonic development [1]. The Polycomb repressive complex 2 (PRC2) is the only known enzyme that mediates di- and tri-methylation of histone H3 on lysine 27 (H3K27Me2/3), modifications believed to be required for PcG-mediated gene repression [2], [3], [4], [5]. PRC2 consist of three core components, Ezh2, Suz12 and Eed, which are all required for early mouse development [6], [7], [8]. H3K27Me3 can function as an epigenetic mark for the recruitment of PRC1, a large heterogenous complex [9], which among others include the Cbx- and Rnf2 (Ring1B) proteins. Rnf2 catalyzes the ubiquitination of histone H2A on lysine 119 (H2AK119Ubi) [10], [11] and as for the members of the PRC2 complex, disruption of the *Rnf2* gene in mouse causes a similar developmental phenotype with arrest at gastrulation [12]. Furthermore, Rnf2 has recently been shown to be part of at least two additional gene regulatory complexes, the E2F6.com-1 complex [13] and the Fbxl10-BcoR complex [14].

The importance of PcG protein complexes in stem cell maintenance and differentiation has been extensively studied in mouse embryonic stem (mES) cells. Previous work have shown that genetic elimination of either PRC1 or PRC2 function, by knockout of *Rnf2* or *Eed* [12], [15], [16], [17], [18] leads to derepression of several lineage-specific genes, that tend to destabilize mES cells, although they still preserve their ability to self-renew and differentiate. Interestingly, the Wutz laboratory showed, that the simultanous loss of both the PRC1 and PRC2 complexes in mES cells abrogates differentiation [19]. In the same study it was suggested that the PRC1- and PRC2-complexes can function independently to repress a common set of genes important for stem cell maintenance.

There are several models explaining how the PcG complexes are recruited to specific target genes. In mammalian cells, it is a long-standing dogma that the PRC2 complex is recruited to target gene promoters by so far unidentified transcription factors (TFs), through the recognition of the underlying DNA sequences. Subsequently, this leads to H3K27 methylation and recruitment of PRC1 through the chromodomain-containing CBX proteins [20], [21]. Several recent publications suggest that Jarid2 can function as a PRC2 recruitment factor [22], [23], [24], [25], [26]. Furthermore, non-coding RNAs (ncRNA) interact with both PRC1 and PRC2 and seem to be working in parallel or in combination with TFs in the ability to recruit the PcG complexes to genomic loci [27], [28], [29]. Interestingly, recent data have implicated ncRNAs as being important for the recruitment of PRC2 to CpG islands and suggested that these genomic entities are sufficient for PRC2 recruitment [30], [31]. In *Drosophila*, several TFs are involved in recruiting the PcG complexes to DNA elements called Polycomb Response Elements (PREs) [32], [33], [34]. However, among these transcription factors only an ortholog of Pho, YY1, is preserved in mammals and has been found to interact with PRC1- [35] and PRC2-subunits [36]. Based on the data from *Drosophila*, it is therefore likely that several TFs beside YY1 are involved in the recruitment of PcG complexes in mammalian cells and identification of such factors is needed in order to define mammalian PREs.

We now present evidence for an interaction between the TF REST (also called Neuron-Restrictive Silencing Factor, NRSF) and the PRC1- and PRC2-complexes, which is independent of RNA. By shRNA mediated knockdown, DNA microarray expression analysis and ChIP analysis, we show that a number of genes are co-regulated by REST and RNF2 in human teratocarcinoma NT2-D1 cells. Genome-wide analysis by ChIP-sequencing (ChIP-seq) in mES cells reveal the co-existence of PRC1 and PRC2 on Rest binding sites. Combining our biochemical interaction analysis of Rest-PcG complexes and the genome-wide analysis of Rest-PcG binding sites, suggest that Rest is required for the recruitment of PRC1 and PRC2 to a subset of its target genes in mES cells. Interestingly, the recruitment of Rnf2 to Rest binding sites can occur independently of both CpG islands and PRC2 activity. Surprisingly, PRC2 was frequently increased around Rest binding sites located in CpG rich regions in the *Rest−/−* mES cells, which suggest that other Rest-associated activities can limit PRC2 recruitment. Based on these observations we propose that Rest has context-dependent functions for PRC1- and PRC2-recruitment to target genes in mammalian cells and that PRC1 is a co-repressor for Rest.

## Results

### REST and the PRC1– and PRC2–complexes interact in mammalian cells

**Figure 1:**
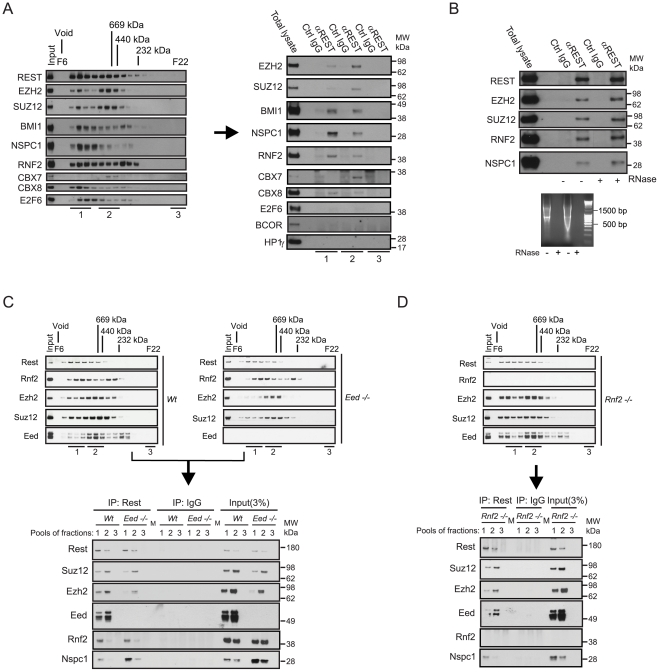
REST and Polycomb Repressor Complex 1 (PRC1) and PRC2 interact *in vivo*. (A) Nuclear extracts from NT2-D1 cells were processed for size-exclusion chromatography followed by Western blotting to reveal the profiles of Polycomb proteins, the transcription factors REST and E2F6. Pooled fractions (1: Fractions F7–9; 2: F11–13; 3: F21–22) were used for immunoprecipitation (IP) with anti-REST or control IgG and processed for Western blotting with antibodies as indicated (total lysate: 12 µg of protein). (B) IPs using REST or control IgG on total nuclear extract (NT2-D1 cells; 500 µg per IP). After IPs the samples were either treated with a combination of RNase V1 and RNase A or left without RNase followed by repeated washes. Eluted proteins were processed for Western blotting using antibodies as indicated. Lower panel: Control experiment for the efficiency of RNase treatment using either 2 µg (left part) or 4 µg (right part) of RNA. Samples were incubated under the conditions used for REST IPs. (C–D) Nuclear protein extracts from mouse embryonic stem cells (mES) of different genetic background (*Wt*, *Eed−/−* or *Rnf2−/−*) were separated by size-exclusion chromatography (C–D: upper panels) and pooled fractions (1: F8–10; 2: F11–13; 3: F22) were processed for IPs (C–D: lower panels) using antibodies for Rest or control IgG. Western blots were processed with antibodies as indicated. Input corresponds to 3% of the material used for each IP. (C) Represents IPs comparing *Wt* and *Eed−/−* mES cells and (D) represents IPs in the *Rnf2−/−* mES cells. The samples were processed for Western blotting with antibodies as indicated. Lanes marked “M” represents loading of a pre-stained molecular weight marker.

We were interested to examine whether the transcription factor REST and the PRC1 complex would interact *in vivo*, encouraged by previous observations, where we identified REST in a double-tag purification of the CBX8 interacting protein, HAN11 (WDR68) (Figure S1; see the information in Procedure S1). We performed size-exclusion chromatography of nuclear extracts from the human teratocarcinoma cells, NT2-D1 (Figure 1A, left part) and HEK 293 cells (Figure S1E) and performed immunoprecipitation of REST from pools of different fractions as indicated (Figure 1A, right part). The data showed, that the PRC1 core subunits, RNF2, BMI1, NSPC1 and CBX8 co-immunoprecipitated with REST from high-molecular weight fractions (F7–9, labelled pooled fractions 1). In addition to CBX8, we also detected CBX7 when performing the REST immunoprecipitation on the pool of fractions F11–13 (labelled pooled fractions 2). Importantly, we did not detect E2F6, BCOR or HP1γ in the REST immunoprecipitations from NT2-D1 cells, showing that the complex that we found associated to REST, contains members of the canonical PRC1 complex and differs from the previously described E2F6.com-1 [13] and Fbxl10-BcoR complexes [14], [37]. For simplicity we will refer to this REST associated PcG complex as PRC1 throughout the remaining part of this work. Importantly, beside core components of the PRC1 complex, we furthermore found that the PRC2 subunits EZH2 and SUZ12 co-immunoprecipitated with REST from pool 1 and 2.

Since it has recently been shown that the long non-coding RNA *HOTAIR* can recruit CoREST/REST/LSD1- and PRC2 complexes through the 5′ and 3′ends respectively [27], we checked whether RNase treatment of our immunoprecipitates would dissociate PcG complexes from REST. As seen in Figure 1B, degradation of single- and double-stranded RNA had no effect on the interactions between endogenous REST, PRC1 and PRC2. Furthermore, we found that the interactions between REST and the Polycomb complexes were not due to DNA bridging, as these were not eliminated by ethidium bromide treatment of immunoprecipitated complexes (Figure S1D). To confirm the interaction between Rest and the PRC1 and PRC2 complexes in mouse embryonic stem (mES) cells, we performed immunoprecipitations of Rest on pooled fractions obtained from size-exclusion chromatography of nuclear extracts from *Wt*, *Eed−/−* and *Rnf2−/−* mES cells (Figure 1C and 1D). Similarly to the results obtained in NT2-D1 cells, we found that Rest interacts with core subunits of the PRC1 and the PRC2 complexes in mES cells. When comparing the relative efficiency of co-immunoprecipitation in *Wt* and *Eed−/−* mES cells, we furthermore found that the interaction between Rest and the PRC2 subunits, Ezh2 and Suz12, did not require Eed. As expected, the total level of the other two core subunits of PRC2, Suz12 and Ezh2, were somewhat reduced in the *Eed−/−* cells (due to destabilization), which was reflected in diminished amounts of these two subunits in the Rest IPs from the *Eed*−/− mES cells. Furthermore, there was a clear change in size distribution of the PRC2 complex in the *Eed−/−* mES compared to the *Wt* cells, showing reduced amounts of the most high-molecular weight forms (Pooled fractions 1, Figure 1C, upper panels). This was reflected in the co-immunoprecipitation of Ezh2 and Suz12 by Rest, where the binding of these two subunits to Rest, in the absence of Eed, was evident in the IP from pool 2, but very reduced in the IP from pool 1 (Figure 1C, lower panel). Moreover, the absence of Eed did not affect the efficiency of co-immunoprecipitation between Rest and the PRC1 subunits, Rnf2 and Nspc1 (Figure 1C). Interestingly, the Nspc1 subunit was more abundant in the *Eed−/−* mES cells (see input lysates), which was also reflected in the Rest IPs when comparing *Wt* and *Eed−/−* mES cells (Figure 1C, lower panel).

Immunoprecipitations performed on fractions from *Rnf2−/−* mES cells furthermore revealed that Rnf2 is not required for the interaction between Rest and the PRC1 subunit Nspc1. This suggested that the interaction between Rest and PRC1 was not mediated directly through Rnf2.

In conclusion, our biochemical data clearly showed that REST interacts with PRC1 and PRC2 protein complexes in mammalian cells and that these REST-PcG complexes are independent of non-coding RNAs.

### REST and PRC1 co-regulate the expression of genes in NT2-D1 cells

**Figure 2:**
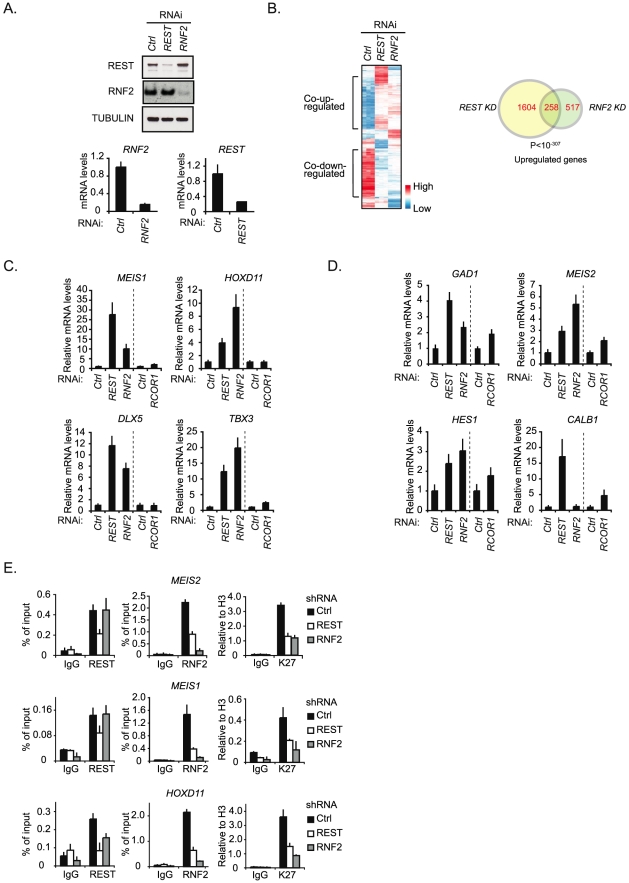
REST is required for PRC1 binding and the maintenance of H3K27Me3. (A) shRNA mediated knockdown of *REST* and *RNF2* in NT2-D1 cells. Samples were processed for Western blot analysis (upper panel) and quantitative real-time RT-PCR (lower panel). (B) Expression array analysis of RNA isolated from NT2-D1 cells treated with a control shRNA or a shRNA against either *REST* or *RNF2*. Left panel: Cluster analysis of regulated genes displayed as a heat-map. Right panel: Venn diagram for genes up-regulated more than 2 fold and a P-value<0.05. In C and D relative mRNA levels in NT2-D1 cells treated with shRNA against *REST*, *RNF2*, or *RCOR1* are shown. Vertical punctured line separate two independent experiments (knockdown of *REST* and *RNF2* were done in parallel and knockdown of *RCOR1* was done separately). (C) Group of genes repressed by REST and RNF2. (D) Group of genes repressed by REST, RNF2 and/or CoREST. (E) ChIP analysis on NT2-D1 cells treated with shRNA against *REST* and *RNF2* using antibodies as indicated (K27 = H3K27Me3). Error bars represent standard deviations calculated from triplicate qPCR reactions.

The interaction between REST and PRC1 suggested that the PRC1 complex might function as a co-repressor for REST. To investigate this, we performed a DNA microarray expression analysis to compare the effect of shRNA mediated knockdown of *REST* with the knockdown of *RNF2* (Figure 2A and 2B). We decided to use the human NT2-D1 cells, since these can easily be induced to differentiate along the neuronal path by retinoic acid (RA) stimulation [38]. As summarized in the cluster analysis and the Venn diagram, a highly significant number of genes were co-regulated by REST and RNF2 (Figure 2B) (see the information on data handling in Procedure S1). While *REST* knockdown lead to significantly increased expression of 1,862 genes (>2 fold), *RNF2* knockdown lead to increase in the expression of 775 genes (>2 fold) (Figure 2B and Table S1). Among these genes, 258 were found to be co-regulated, corresponding to more than 30% of all the genes up-regulated in response to *RNF2* knockdown. Although, the majority of the co-regulated genes were up-regulated, we also found that a considerable number of genes were co-down-regulated, suggesting that we not only observe direct effects, but also indirect effects on expression. In agreement with previous reports on REST- and PcG targets, co-up-regulated genes enrich for developmental functions, while co-down-regulated genes showed no significant enrichment for any particular biological function (Table S9). This suggests that among the co-up-regulated genes, there was enrichment for genes that were directly targeted by the REST-PRC1 complex.

To compare the influence of RNF2 to a well-described REST-interacting co-repressor complex, the CoREST complex [39], we decided to perform a new shRNA mediated knockdown experiment using constructs for *REST*, *RNF2* and *RCOR1* (gene coding for the CoREST protein) (Figure 2C and 2D). Using QPCR, we analyzed the change in expression of 7 different genes from the group of 258 co-regulated genes identified in the microarray (Figure 2B). Interestingly, the data suggest that the genes can be divided into two groups. The first group contains genes (*MEIS1*, *HOXD11*, *DLX5* and *TBX3*) that were co-regulated by REST and RNF2 but only slightly affected by *RCOR1* knockdown (Figure 2C). The second group includes genes (*GAD1*, *MEIS2* and *HES1*) that were co-regulated by REST and RNF2, but furthermore showed an increase in expression upon *RCOR1* knockdown (Figure 2D). We also found an example of a gene (*CALB1*) that was co-regulated by REST and CoREST, but unaffected by *RNF2* knockdown (Figure 2D).

To confirm the interaction between REST and RNF2 on co-regulated target genes we performed ChIP for two of the genes in the first group (*MEIS1* and *HOXD11*) and one gene in the second group (*MEIS2*) in NT2-D1 cells treated with shRNAs for *REST*, *RNF2* or empty control shRNA (Figure 2E). As expected, *REST* knockdown reduced the level of REST at all three target genes. Importantly, *REST* knockdown also lead to a reduced amount of RNF2 at these genes (Figure 2E). In contrast, while a shRNA against *RNF2* almost completely eliminated RNF2 binding to the *MEIS1* and *MEIS2* genes, it had no effect on REST binding, which is in agreement with REST being a DNA binding factor recruiting RNF2. At the *HOXD11* locus there was a small, but significant, reduction of REST binding in response to RNF2 knockdown. Interestingly, H3K27Me3 was significantly reduced in both *REST-* and *RNF2* knockdowns, indicating that the PRC2 complex interacted with both REST and RNF2 containing complexes on the *MEIS1*, *MEIS2* and *HOXD11* genes.

Altogether we found that REST was required for the recruitment of RNF2 and the maintenance of H3K27Me3 on a selected number of neuronal genes and that REST and RNF2 co-regulate a highly significant number of genes in NT2-D1 cells.

### Rest is required for the recruitment of PRC1 and PRC2 to specific genes in mES cells

**Figure 3:**
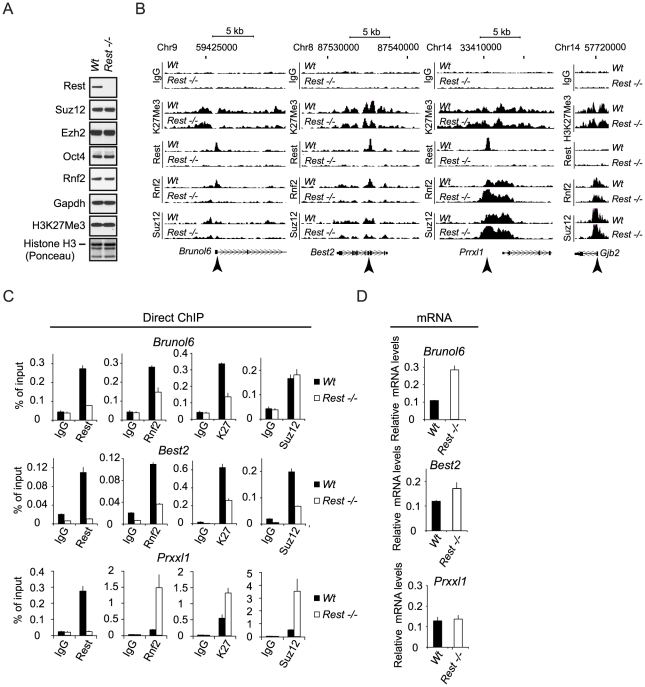
Rest regulates recruitment of PRC1 and PRC2 at specific genomic loci. (A) Western blot analysis of *Wt* and *Rest*−/− mES cell lysates. Histones were extracted using LSB. (B) ChIP-seq profiles for Rnf2, Suz12 and H3K27Me3 levels in *Wt* and *Rest−/−* mES cells for three Rest target genes (*Brunol6*, *Best2* and *Prrxl1*) and as control, one Polycomb target gene with no Rest binding site (*Gjb2*). Direct ChIP for the three Rest target genes shown in (B) comparing *Wt* and *Rest−/−* mES cells (primer positions are indicated by arrow heads in B and K27 = H3K27Me3) and mRNA levels (C). Error bars represent standard deviations calculated from triplicate qPCR reactions.

To test if Rest is required for the recruitment of PRC1 and PRC2 to their target genes in mouse embryonic stem (mES) cells, we performed our analyses in the previously published *Wt* and *Rest*−/− mES cells [40], [41]. We observed that the overall levels of Suz12, Ezh2, Rnf2 and Oct4 proteins were unchanged in the *Rest−/−* mES cells as compared to the *Wt* mES cells (Figure 3A). Although, the global level of the H3K27Me3 was unchanged between the *Wt* and *Rest*−/− mES cells (Figure 3A), ChIP-sequencing (ChIP-seq) (see information on data handling in Procedure S1) for H3K27Me3, Suz12 and Rnf2 revealed clear local differences for these factors. This is illustrated in Figure 3B by ChIP-seq profiles for three genes (*Brunol6*, *Best2 and Prrxl1*) showing co-localization between Rest, Rnf2 and Suz12 in the *Wt* mES cells. Notably, for two of the genes (*Brunol6* and *Best2*) there was an almost complete loss of Rnf2 binding at the center of the Rest peaks in the *Rest*−/− mES cells. For the *Prrxl1* gene, there was a surprising increase in Rnf2 binding, suggesting that Rest can also counteract PRC1 recruitment at certain loci. Moreover, the increase in Rnf2 was accompanied by an increase in PRC1 subunit Cbx7 on the Prrxl1 gene (Figure S2C). The effects on Suz12 binding were similar, but less pronounced, with reduced binding at *Brunol6* and *Best2* and somewhat increased binding at the *Prrxl1* gene. For the distribution of H3K27Me3, we observed more widespread effects and the signals were reduced throughout the gene body at *Brunol6* and *Best2*, while at *Prrxl1* the signal was essentially unchanged. Moreover, the non-Rest target gene, *Gjb2*, did not show changes in Rnf2 and Suz12 binding as well as H3K27Me3 levels in the *Rest−/−* mES cells (Figure 3B). Importantly, the mRNA levels for *Brunol6* and *Best2* were up-regulated in *Rest*−/− mES cells while unchanged for *Prrxl1* (Figure 3D), showing a functional link between the loss of PcG proteins, the reduced H3K27Me3 mark and gene activity in the absence of Rest. We confirmed the effects observed in the ChIP-seq profiles by direct ChIP (Figure 3C) using primers at the Rest peak position (as indicated by arrow heads in Figure 3B).

### Global analysis of Rest and PcG occupancy in *Wt* and *Rest−/−* mES cells

**Figure 4:**
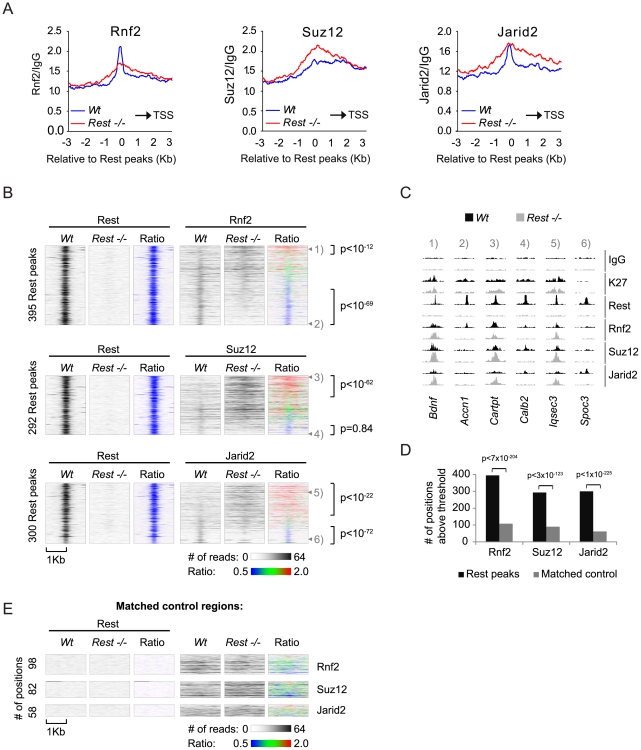
Occupancy of PRC1 and PRC2 at Rest peaks in *Wt* and *Rest*−/− mES cells. (A) Average ChIP-seq read counts in *Wt* control and *Rest*−/− mES cells, normalized to IgG controls for Rnf2, Suz12 and Jarid2 within 3 Kb of the 3,378 peaks identified for Rest in the *Wt* control cells. Black arrow indicates the direction of the nearest TSS. (B) Heat-maps showing Rnf2, Suz12 and Jarid2 occupancy in a 2 Kb window centered around individual Rest peaks identified in *Wt* control mES cells. The ratiometric heat-maps depict the part of the profiles that were increased, unaffected, or decreased in *Rest−/−* mES cells as red, green and blue, respectively. Out of the 3,378 Rest peak positions identified in the *Wt* control mES cells, positions with very low levels or no PcG (Rnf2, Suz12 and Jarid2) ChIP-seq signal in either *Wt* control or *Rest−/−* mES cells were filtered out (for thresholds and genomic peak positions see Table S3). The regions were sorted according to the fold change in PcG (Rnf2, Suz12 and Jarid2) signal between the *Wt* control and *Rest−/−* mES cells, within the Rest peaks. P-values were calculated by comparing the number of Rest binding sites with 1.5 fold increase or decrease to those expected from a population with similar distribution as the matched control using chi^2^-tests (C) ChIP-seq profiles for the positions marked 1 to 6 in Figure 4B as examples. Data from *Wt* and *Rest−/−* mES cells are shown in black and grey, respectively (K27 = H3K27Me3). (D) Diagram showing the number of Rest peaks and matched control regions (no Rest peaks) scored positive for Rnf2, Suz12 or Jarid2 signal. The filtering process described in the legend for Figure 4B. P-values compares binding sites scoring positive for PcG binding to those that scored positive by chance in the matched control regions using chi^2^-tests (E) Heat-maps of the matched control regions shown in 4D.

To obtain a global picture of how important Rest is for the recruitment of PRC1 and PRC2 in mES cells, we plotted the average distributions of Rnf2 (PRC1) and Suz12 (PRC2) signals in *Wt* and *Rest*−/− mES cells from our ChIP seq analysis relative to 3,378 identified Rest binding sites in the *Wt* cells (Figure 4A and Table S2). We included Jarid2 in this analysis, since this protein has recently been shown to be important for PRC2 recruitment in mES cells [24], [25], [22], [23], [26]. These results showed that in the *Wt* mES cells Rnf2, Suz12 and Jarid2 all enrich at Rest binding sites. Interestingly, Rnf2 and Jarid2 displayed focused localization at the Rest binding sites, whereas Suz12 had a broader distribution. When comparing *Rest*−/− to *Wt* mES cells we observed that the overall Rnf2 signal was reduced at the center of the Rest peaks, but was slightly increased in the flanking regions (Figure 4A). Surprisingly, the overall signal for Suz12 was clearly increased in the *Rest*−/− mES cells, suggesting that Rest can function to limit PRC2 binding in certain contexts. Moreover, when looking at these average distributions, we observed a similar increase in the Jarid2 signal flanking the Rest binding sites in the *Rest*−/− mES cells (Figure 4A), which is in agreement with Jarid2 being part of the PRC2 complex. As we observed reduced Rnf2 and Suz12 levels at specific Rest binding sites in the *Rest−/−* mES cells (Figure 3B and 3C), we wanted to investigate how frequently Rest target loci had lost or gained PcG-binding. For this we generated heat-maps based on ChIP-seq tracks for Rnf2, Suz12 and Jarid2 around individual Rest binding sites scored positive for PcG proteins either in *Wt* or *Rest−/−* mES cells (Figure 4B and Table S3). These heat-maps showed that Rnf2, Suz12 and Jarid2 all displayed a focused signal centered at a large fraction of the Rest binding sites in *Wt* mES cells, whereas this focused signal was absent in the *Rest*−/− mES cells. To test if the PcG binding was specific for Rest binding sites, we generated matched control regions for all 3,378 Rest binding sites identified in *Wt* mES cells. Matched control regions were randomly chosen, but matched to the distribution of the Rest binding sites relative to TSS [42]. In agreement with the conclusion that Rnf2, Suz12 and Jarid2 enrich at Rest binding sites, we found roughly four to five times as many Rest binding sites scored positive for PcG proteins compared to the matched control regions (Figure 4D and 4E). Moreover, at the 395 Rest binding sites shown for Rnf2, the signal was reduced 1.5 fold or more at 172 sites (Ratio-image: blue) and increased 1.5 fold or more at 45 sites (Ratio-image: red) in the *Rest*−/− mES cells compared to the *Wt* mES cells (Figure 4B; upper part). Examples of ChIP-seq tracks for target genes from upper and lower parts of the heat-maps are shown in Figure 4C (numbers 1–6 refers to the position in the individual heat-maps). For Suz12, we found that out of 292 Rest binding sites 45 and 105 sites had a signal that was reduced or increased 1.5 fold or more, respectively (Figure 4B; middle part). For Jarid2, we found that out of 300 Rest binding sites, the signal was reduced 1.5 fold or more at 85 sites and increased 1.5 fold or more at 152 sites (Figure 4B; lower part). Interestingly, heat-maps furthermore showed that the loss of binding in the *Rest*−/− mES cells, for all three proteins (Rnf2, Suz12 and Jarid2) was focused at the Rest binding site. On the other hand, increased binding was more widely distributed and not confined to the actual Rest binding site (Figure 4B). This explains why we observed increased signals for PcG proteins in the regions flanking the Rest binding sites in Figure 4A. When the changes in PcG binding observed at Rest binding sites were compared to the changes at the matched control regions, we found that both the loss and the gain of signal observed at Rnf2- and Jarid2-positive Rest binding sites, as well as the gain of signal at Suz12-positive Rest binding sites, occurred much more frequently than would be expected from Rnf2-, Jarid2-, and Suz12-positive matched control regions (Figure 4B and 4E).

In conclusion, we found that Rest was required for the specific recruitment of Rnf2 to a considerable number of Rest binding sites, whereas Suz12 and Jarid2 showed dependency on a smaller number of loci. In addition, many loci in the *Rest*−/− mES cells, which bound Rest in the *Wt* mES cells, had increased levels of Suz12 and Jarid2. This was also observed for Rnf2, but less frequently, suggesting that Rest can also limit PcG protein recruitment and that other factors control how Rest affects the level of PRC1 and PRC2 at specific genomic loci.

### Rest recruits PRC1 independently of CpG islands

**Figure 5:**
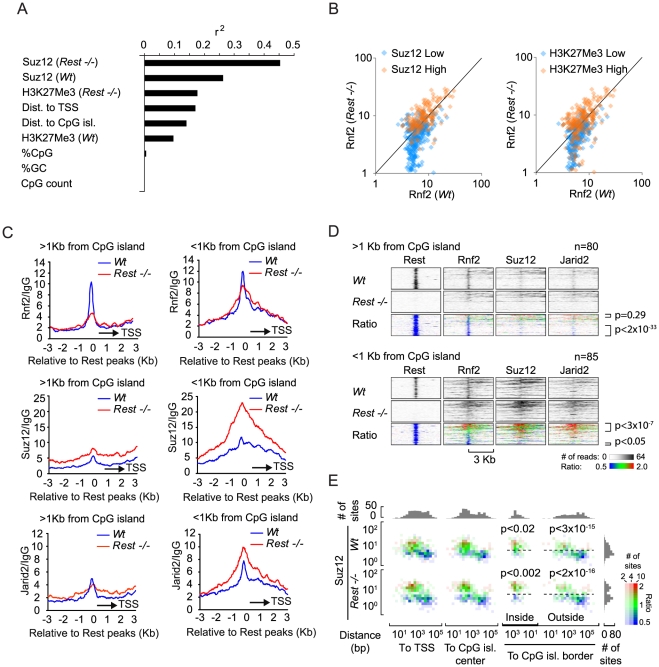
Effects of different parameters on Rnf2 binding. (A) Graph showing the Pearson's correlation coefficients (r^2^) of the change in Rnf2 at Rest peaks correlated to different parameters as indicated (the data were log-transformed before the correlation was done). (B) Scatter diagram showing the Rnf2 signal for the Rest peaks with either relative low (blue color) or high (red color) levels of Suz12 (left panel) or H3K27Me3 (right panel) in the *Wt* versus *Rest−/−* mES cells. The Rnf2 positive Rest peaks shown in Figure 4B were separated into two groups having either relative low (blue color: <5 reads on average within 1 Kb of the Rest peak) or relative high (red color: >5 reads on average within 1 Kb of the Rest peak) ChIP-seq signals for Suz12 and H3K27Me3. (C) Average ChIP-seq read counts for Rnf2, Suz12 and Jarid2 in *Wt* and *Rest*−/− mES cells normalized to IgG controls. The regions were subdivided into two groups, depending on whether or not a CpG island was located within 1 Kb of the borders of the Rest peaks. Only regions with a direct overlap between Rest and Rnf2 peaks in the *Wt* control mES cells were included (Table S4). (D) Heat-maps of Rnf2, Suz12 and Jarid2 for the individual peak positions analysed in C. The regions were sorted according to the fold change in PcG (Rnf2, Suz12 and Jarid2) signal between the *Wt* control and *Rest−/−* mES cells, within the Rest peaks. The ratiometric region map depicts the part of the profiles that were increased, unaffected, or decreased in *Rest−/−* mES cells as red, green and blue, respectively. (E) Color-coded density plots showing the difference in Rnf2 ChIP-seq signal in *Wt* and *Rest−/−* mES cells relative to the distance to TSS, distance to the nearest CpG-island, and the Suz12-signal at the 395 Rest peak positions that were scored Rnf2-positive. Densities, within each bin, depend on the number of peaks, whereas the color-coding corresponds to the average Rnf2*_Rest−/−_*/Rnf2*_Wt_* ratio with red, green and blue illustrating increased, unaffected, or decreased signal, respectively. Peaks were binned in 1.5 fold bins for Suz12 and 2 fold bins for the TSS or CpG position. The flanking histograms in the top and right part of the figure illustrate the number of peaks in each bin. P-values were calculated using heteroschedastic Student's t-tests.

Given the biochemical interaction between Rest and Rnf2 and the enrichment of Rnf2 at Rest binding sites, it was surprising to see that Rest binding sites in *Rest−/− mES* cells, not always lost Rnf2 binding, but that some Rest binding sites even gained Rnf2 signal. To study this enigma further, we correlated the changes in Rnf2 binding between *Wt* and *Rest−/−* mES cells to several different parameters, such as PRC2 levels and distance to the nearest CpG island (Figure 5A). This analysis showed a strong correlation between changes in Rnf2 binding and the levels of Suz12 at Rest binding sites in both *Wt* and *Rest−/−* mES cells (Figure 5A). Changes in Rnf2 binding also correlated well with the distance to TSS and CpG islands, but not to the overall composition (% CpG or %GC) and size of the nearest CpG island (Figure 5A).

To analyze the PRC2 dependency for PRC1 recruitment further, we separated Rnf2-positive Rest binding sites into two groups, which either had relatively high or low Suz12 or H3K27Me3 signals in the *Wt* mES cells. The signal for Rnf2 in *Wt* versus *Rest−/−* mES cells was plotted in a scatter diagram (Figure 5B). The results showed that Rnf2 binding was more likely to be Rest-dependent at sites with low Suz12 and low H3K27Me3 levels (Figure 5B; blue color) compared to sites with high signals for Suz12 and H3K27Me3 in *Wt* mES cells (Figure 5B; orange color). In contrast, sites with high levels of Suz12 and H3K27Me3 were more likely to maintain or even gain Rnf2 binding in the absence of Rest. Furthermore, we observed that sites with increased PRC2 binding in general had increased Rnf2 levels in *Rest−/−* mES cells (Figure S3).

To study the effect of CpG islands on changes in Rnf2 binding in the absence of Rest, we separated a subset of Rest binding sites with the strongest Rnf2 binding (*Wt* mES) into two categories: 1) sites with an annotated CpG island within 1 Kb (CpG island-positive) and 2) sites more than 1 Kb away from any annotated CpG island (CpG island-negative) (UCSC browser). Using this approach we found that out of 165 Rest binding sites overlapping with Rnf2 sites app. 50% are within 1 Kb of a CpG island (Figure 5C, 5D and Table S4). Interestingly, when we plotted the average distribution of Rnf2 at CpG island-negative Rest binding sites (>1 Kb from a CpG island), we observed that the Rnf2 enrichment was almost completely lost at Rest binding sites in the *Rest*−/− mES cells. In contrast, at CpG islands-positive Rest binding sites (<1 Kb from a CpG island), we observed only a minor decrease in Rnf2 binding (Figure 5C and 5D). For Suz12, the data showed that the levels of Suz12 were clearly higher at CpG islands-positive Rest binding sites, and that the absence of Rest resulted in a marked increase in Suz12 at these sites (Figure 5C and 5D). We found similar effects for Jarid2 (Figure 5C and 5D), suggesting that CpG islands could be part of a mechanism responsible for the increased recruitment of PRC2 in the absence of the transcription factor Rest in the mES *Rest−/−* mES cells.

Finally, we wanted to distinguish, whether the effect of CpG islands on Rnf2 binding was due to the presence of PRC2 at the CpG islands, or if CpG islands represented an independent entity responsible for Rnf2 recruitment. Therefore, we plotted the Rnf2*_Rest−/−_*/Rnf2*_Wt_* ratio of Rnf2-positive Rest binding sites relative to the position of the nearest TSS or the nearest CpG island, as well as to the level of Suz12 in either *Wt* or *Rest−/−* mES cells. As previously described, we observed that a larger fraction of Rnf2 positive Rest binding sites had increased Rnf2*_Rest−/−_*/Rnf2*_Wt_* ratio at positions close to TSS and CpG islands (Figure 5E, red coloring), compared to those that were located far from TSS or CpG islands (Figure 5E, blue coloring). Importantly, the key determinant for the change in Rnf2*_Rest−/−_*/Rnf2*_Wt_* ratios appeared to be the level of the PRC2 subunit Suz12. Rest binding sites with high Suz12 levels and located at or close to CpG islands, had significantly increased Rnf2*_Rest−/−_*/Rnf2*_Wt_* ratios, whereas those with low Suz12 levels had a lower Rnf2*_Rest−/−_*/Rnf2*_Wt_* ratio (Figure 5E). Also, a minor group of Rnf2-positive Rest binding sites, far from annotated CpG islands, had a high level of Suz12 and these had a correspondently increased Rnf2*_Rest−/−_*/Rnf2*_Wt_* ratio compared to sites with similar position and lower Suz12 levels. In summary, it appears from this analysis that 1) Rest was the dominant recruitment factor for PRC1 at Rest binding sites with low PRC2 levels, and 2) that the increased recruitment of Rnf2 observed at CpG islands in *Rest−/−* mES cells was an indirect effect of increased PRC2 recruitment to CpG islands and TSS-proximal Rest binding sites in the *Rest−/−* mES cells.

### Recruitment of PRC1 to Rest binding sites, independent of Eed and the H3K27Me3 mark

**Figure 6:**
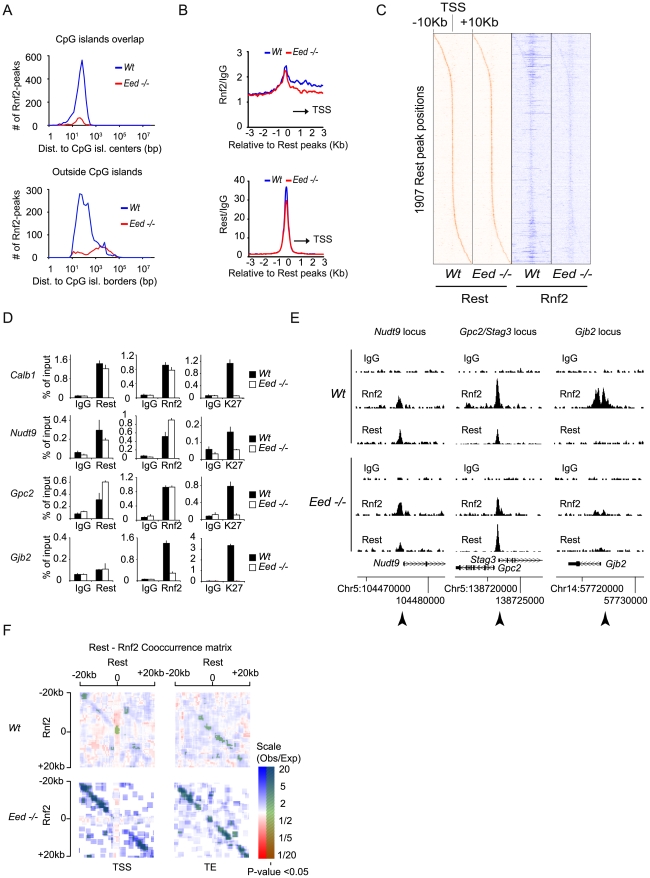
The recruitment of PRC1 at a number of Rest binding sites is independent of PRC2 activity in mES cells. (A) Histograms showing the number of Rnf2 peaks in *Wt* and *Eed*−/− mES cells at positions relative to CpG islands. Upper panel: Rnf2 peaks that overlap with CpG-islands relative to the distance between the center of the peak and the center of the CpG-island. Lower panel: Rnf2 peaks, not overlapping with annotated CpG-islands, plotted relative to the distance between the borders of the Rnf2 peak and the border of the CpG-island. (B) Average ChIP-seq reads in E14 *Wt* and *Eed*−/− for Rnf2 (upper panel) and Rest (lower panel) within 3 Kb of the 5,097 Rest peaks identified in E14 *Wt* mES cells (Table S5). Read counts were normalized to the IgG control. The black arrow indicates the direction of the nearest TSS. (C) Heat-maps of Rest and Rnf2 in *Wt* and *Eed*−/− mES cells at 1,907 Rest peaks within 10 Kb of a TSS. (D) Direct ChIP in E14 *Wt* and *Eed*−/− mES cells for *Calb1*, *Nudt9*, *Gpc2/Stag3* and *Gjb2* (positions of primers used are indicated by arrow heads in E). Error bars represent standard deviations calculated from triplicate qPCR reactions. (E) ChIP-seq profiles for the *Nudt9, Gpc2/Stag3 and Gjb2* loci using antibodies for Rnf2, Rest and control IgG. (F) Co-occurrence matrix of Rest and Rnf2 peaks from *Wt* and *Eed−/−* mES cells. The maps are based on scans, with a 2,500 bp window, along different positions near the transcription start site (TSS) or transcription end points (TE) annotated in the mouse genome (Mouse genome: mm9). For each scan the number of observed peaks were calculated and normalized to the number of expected peaks based on the frequency of Rest and Rnf2 peaks in that particular position, relative to the TSS or TE. Scans with a higher or lower frequency than expected from random localization, were colored blue or red, respectively. Scans with a P-value below 0.05 (Bonferroni corrected for multiple sampling), are overlaid with transparent green color.

The general view that PRC1 is recruited to chromatin exclusively by PRC2 activity [43] has recently been challenged, by the observation that mES cells lacking PRC2 activity still maintained H2AUbi marked histones catalyzed by the PRC1 complex [19]. Since we observed that Rest was needed for PRC1 recruitment at Rest binding sites with low levels of Suz12 and H3K27Me3 (Figure 5B), we wanted to test if Rest could recruit PRC1 to Rest binding sites in cells deficient for PRC2 activity. We performed ChIP-seq for Rest and Rnf2 in E14 *Wt* control and *Eed−/−* mES cells and found that app. 90% of the Rnf2 binding sites identified in the *Wt* cells were lost in *Eed−/−* cells (Table S6). In line with PRC1 being recruited to CpG islands via PRC2, we observed that the loss of Rnf2 binding sites were most pronounced for peaks positioned at or close to CpG islands (Figure 6A, upper part), while the majority of binding sites positioned distant from CpG islands were in general maintained in the *Eed−/−* mES cells (Figure 6A, lower part). Nonetheless, the average Rnf2 signal at Rest binding sites was only marginally reduced (Figure 6B, upper part) when analyzing all Rest binding sites both distant from CpG islands and at CpG island proximal regions. Notably in the *Wt* mES cells, we observed increased Rnf2 binding from the Rest binding sites towards the TSS, which was virtually lost in the absence of Eed (Figure 6B, upper part). Furthermore, when comparing the distribution of Rest peaks in *Wt* versus *Eed*−/− mES cells, we only observed a minor effect indicating, that the overall binding of Rest to DNA was independent of PRC2 (Figure 6B, lower part).

In order to visualize the data summarized by the average distributions in Figure 6B, for individual genes, we generated heat-maps based on ChIP-seq tracks for individual Rest binding sites within 10 Kb of a TSS (Figure 6C). In agreement with the data in Figure 6B, the wide-spread Rnf2 signals in the *Wt* was virtually lost in the *Eed*−/− mES cells, but the signal was clearly maintained at the Rest binding sites (Figure 6C).

To confirm the results obtained by ChIP-seq, we performed direct ChIP on four genes. We compared the Rnf2 binding at Rest binding sites in *Wt* and *Eed*−/− cells and also included a ChIP for the H3K27Me3 mark. As shown in Figure 6D, the four genes behaved differently. *Gjb2*, a PcG target gene [44] showed very low levels of Rest binding in the *Wt* mES cells, which was not affected in the *Eed*−/− mES cells, while H3K27Me3 was absent and Rnf2 strongly reduced. For the *Calb1* gene, a classical Rest target [45], we confirmed a strong Rest enrichment, which correlated with enrichment for Rnf2 in the *Wt* mES cells (Figure 6D). Even though H3K27Me3 was absent at the *Calb1* locus in the *Eed*−/− mES cells the binding of Rnf2 was preserved (Figure 6D), demonstrating PRC2- and H3K27Me3-independent recruitment of Rest-PRC1. *Nudt9* and *Gpc2*, two other Rest targets in mES cells showed either a small decrease or increase, respectively, in Rest binding in the *Eed−/−* mES cells as compared to the *Wt* mES cells, while Rnf2 was not reduced. Similarly, we detected Cbx7 binding on two non-Rest PcG targets (*Gjb2* and *Neurog1*) in *Wt* mES cells that was lost in *Eed−/−* mES cells, while Cbx7 was still bound to the *Stag3* gene in *Eed−/−* mES cells (Figure S2). In Figure 6E we present ChIP-seq profiles for the *Nudt9*, *Gpc2/Stag3*, and *Gjb2* loci, which reflects the results obtained by direct ChIP (ChIP followed by QPCR analysis; arrow heads indicate the position of primers used in Figure 6D). It is noteworthy that not all Rest binding sites were preserved between the two Wt mES cell lines (compare Figure 3B and Figure 6E to Figure S4).

Since both Rnf2 and Rest binding sites in E14 *Wt* mES cells frequently appeared in close proximity of the TSS of genes, we asked whether Rest and Rnf2 co-localized more frequently than expected by chance in *Wt* and *Eed*−/− mES cells. We calculated the frequencies of Rest binding sites alone, Rnf2 sites alone or in combination with Rest, 20 Kb up- and down-stream of all annotated TSS positions and transcription ends (TE) in the mouse genome (mm9). We plotted ratios between the observed frequencies versus the frequencies expected by chance in a co-occurrence matrix (Figure 6F). The observed co-occurences of Rest and Rnf2 at the TSS in *Wt* mES cells were actually significantly below what could be expected by chance (red), whereas upstream and downstream of TSS and at TE, we observed significantly more co-occurrence than expected by chance (blue) (Figure 6F). Remarkably and in agreement with Rnf2 binding being preserved at Rest binding sites in the *Eed*−/− mES cells, we observed much higher co-occurrence than expected by chance both up- and down-stream of the TSS and TE in the absence of PRC2 activity.

Taken together these results substantiate the relation between PRC1 and Rest in mES cells and suggest that Rest-PRC1 can be recruited to neuronal genes independently of the PRC2 complex.

### Neuronal differentiation of NT2-D1 cells

**Figure 7:**
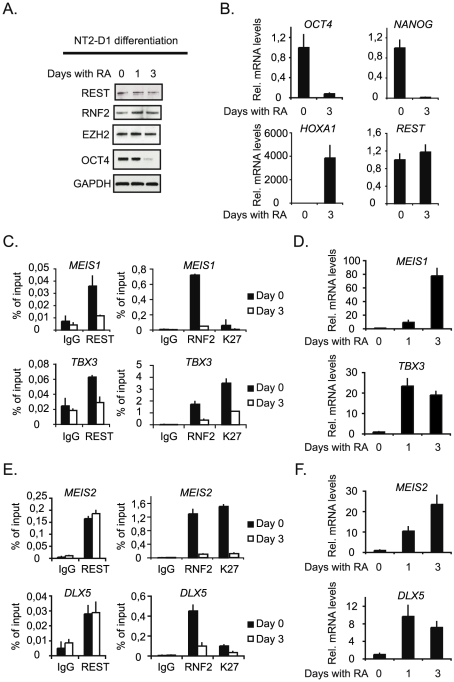
Neuronal differentiation in NT2-D1 cells. NT2-D1 cells were treated with Retinoic Acid (RA) for 1 and 3 days or left untreated (day 0). Protein-, RNA- and ChIP-samples were harvested. (A) Western blot analysis of protein samples taken at day 0, 1 and 3. (B) Relative mRNA levels at day 0 and 3 of *REST*, *OCT4* and *NANOG* and *HOXA1*. (C) Group of genes, which loose binding of both REST and PcG proteins during differentiation. ChIP analysis was performed for day 0 and 3 of RA treatment. (D) Relative mRNA levels of the genes displayed in (C), for untreated cells and cells treated with RA for 3 days. (E) Group of genes, which maintain REST binding but loose PcG proteins during differentiation. ChIP analysis at day 0 and 3 after RA treatment. (F) Relative mRNA levels of the genes displayed in (E) after 0 and 3 days of RA treatment. Error bars represent standard deviations calculated from triplicate qPCR reactions.

Finally, we wanted to study the interplay between REST and the PcG complexes at genes that are induced during differentiation. In order to do this we took advantage of the fact that NT2-D1 cells can be induced to differentiate along the neuronal path by retinoic acid (RA)- stimulation, and used these cells to study the dynamics of REST and RNF2 binding on a number of selected target genes that were co-regulated by REST and RNF2 (Figure 2B). RA-stimulation for 3 days led to an almost complete loss of the pluripotency factor OCT4 (Figure 7A), while the protein levels of EZH2, RNF2 and REST were only marginally affected. Furthermore, *OCT4* and another pluripotency factor *NANOG* were almost completely silenced transcriptionally (Figure 7B), while the *REST* transcript was unaffected and *HOXA1* was strongly induced (Figure 7B). The genes we selected for ChIP analysis were all induced by RA-stimulation (Figure 7D and 7F), but can be divided into two groups, based on the events taking place at the REST binding sites. The first group is represented by *MEIS1* and *TBX3*, which both have reduced REST binding to the gene after 3 days of RA-stimulation (Figure 7C). These genes lost RNF2 binding and had reduced H3K27Me3 after RA treatment. For the second group of genes represented by *MEIS2* and *DLX5*, REST stayed on the genes after 3 days of RA-stimulation, while RNF2 was lost (Figure 7E). These genes also lost H3K27Me3 (*MEIS2*) or had significantly reduced H3K27Me3 after RA-stimulation (*DLX5*). The data suggested, that gene induction in response to RA-stimulation does not necessary require displacement of REST, but correlated with the displacement of PRC1 (RNF2) and reduction in H3K27Me3. The determining factor(s) for how REST-PcG complexes are regulated at specific target genes is still an open question. We imagine that post-translational modifications of REST and/or PcG proteins could be part of a mechanism regulating the interaction(s) between these factors and the affinity of REST for DNA. Furthermore, other REST associated factors, beside the PcG proteins, could affect the affinity of REST complexes for chromatin on individual genomic loci.

## Discussion

In *Drosophila* there are at least 5 different transcription factors shown to be important for PcG recruitment [32], however, among these only the ortholog of Pho called YY1 is preserved in mammals. Even though YY1 and a second transcription factor, E2F6, have been found to interact with PRC2 [36], [46] and PRC1 in mammals [13], [14], [35], [47], there have been only two studies presenting evidence to support the existence of mammalian PREs. The first potential mammalian PRE identified was an element shown to regulate the expression of a hindbrain segmentation gene *MafB* (*Kreisler*). This putative PRE was shown to recruit PRC1 and PRC2, thereby mediating silencing of an ectopically introduced transgene in both flies and mice [48]. In a second study Woo et al. found a 1.8 Kb element between the *HOXD11* and *HOXD12* genes that was regulated by PcG proteins and H3K27Me3 [49]. Interestingly, this locus contained several YY1 binding sites and YY1 binding to the locus was coincident with PcG enrichment. Loss of YY1 binding sites had however only modest effect on repression.

Based on previous studies showing H3K27Me3 enrichment around REST binding sites (RE1 elements) in human T-cells [50] and the fact that we identified REST in the purification of the CBX8 interacting protein HAN11 ([44] and Figure S1), we decided to investigate the functional relationship between REST and the PRC1- and PRC2-complexes.

We found that REST was in complex with core members of both the PRC1- and PRC2-complexes in the human NT2-D1, HEK293 cells and mouse embryonic stem cells. For PRC2 we found that REST interacted with the three essential core members: EZH2, SUZ12 and EED, showing that REST was associated to a functional PRC2 complex. Due to the existence of both PRC1 and other RNF2 containing complexes, we performed a more detailed analysis of the REST-PRC1 complex. This analysis revealed that REST form complex with RNF2 and other selected core members of the PRC1 complex (BMI1, NSPC1, CBX7 and CBX8). Importantly, we did not detect E2F6, HP1γ or BCOR in the REST IPs, proteins that have previously been described in RNF2 containing E2F6.com-1 (E2F6 and HP1γ) [13] and Fbxl10-BcoR (BcoR and HP1γ) [14], [37] complexes. These observations suggest that the REST-PRC1 complex that we have identified is distinct from the previously described Rnf2-containing complexes.

Recently, an interesting work related to PRC2 and Rest was published by Tsai et al. [27]. They found that the previously described long non-coding RNA, *HOTAIR* [51], [52] interacted not only with PRC2 through its 5′end, but even bound a LSD1/CoREST/REST-complex through the 3′end and in this fashion function as a modular bifunctional RNA. To understand if *HOTAIR* or other ncRNAs could be involved in bridging PRC1 and PRC2 to REST in the complexes that we identified, we treated our REST immunoprecipitates from NT2-D1 cells with RNases, but did not observe changes in the relative amounts of PRC1 and PRC2 subunits bound to REST. Thus, we conclude that the REST-PcG complexes that we have identified are stable in the absence of ncRNAs such as *HOTAIR*. Further studies are needed to determine if PRC1 and PRC2 are part of one and the same REST-PcG complex or are separate entities.

In support of a functional interaction between REST and PRC1, we found a significant number of genes that were co-regulated in the NT2-D1 cells. In line with REST and PRC1 being repressors of gene activity, the majority of co-regulated genes were up-regulated (258) and gene-ontology analysis revealed an enrichment of genes involved in development and neuronal function (Table S9). This is in agreement with previous publications showing that REST and PRC1 directly target genes involved in developmental processes and neuronal function. In contrast, the group of co-down-regulated genes did not enrich for any particular biological function suggesting that this group of genes were not primary targets of REST and PRC1.

In our genome-wide analysis of Rest and PcG occupancy in mES cells, we furthermore found that both PRC1 (Rnf2) and PRC2 (Suz12, Jarid2) subunits specifically enriched at Rest binding sites. Similarly to the co-up-regulated genes identified in NT2-D1 cells, there was enrichment for genes involved in developmental and neuronal function among the genes targeted by both Rest and the Polycomb complexes (Figure S5 and Table S7) suggesting a similar set of target genes in these different cell types of human (NT2-D1 cells) and mouse origin (mES cells). However, the loss of REST in NT2-D1 and mES cells have different outcome with respect to transcription. Whereas, loss of REST in NT2-D1 cells leads to up-regulation of a large group of genes (1862) the effect in mES cells was less than expected and did not compare to the number of target genes [41] (Tables S1 and S2). This suggest that genes bound by REST in NT2-D1 and mES cells are differently regulated and that in mES cells, Rest target genes are regulated by additional repressive mechanisms, which ensure that developmental genes are kept in an “off-state”.

The genome-wide analyses for PcG occupancy in mES cells, demonstrated a specific enrichment of the average Rnf2, Suz12 and Jarid2 signals at the positions corresponding to Rest binding sites (Figure 4A and Figure 6B). To improve the validity of these observations, we included a range of controls and extra analyses. *Bona fide* signals may co-exist at specific genes or gene features in a manner that seems significant due to congregations at groups of genes or gene features. Therefore, we used a set of randomly chosen control positions that matched the Rest-binding sites in terms of TSS-proximity. In comparison, Rest binding sites scored positive for PcG proteins (Rnf2, Suz12 and Jarid2) 4–5 times as frequent as the matched control regions (Figure 4D). Co-occurrence analysis of Rest and Rnf2 furthermore allowed us to rule out that Rest and Rnf2 co-enriched by chance because both proteins were frequently found at TSS (Figure 6F). With these tests, we are confident that the co-localization of Rest and PcG complexes in the genome was unlikely to be a mere coincidence. In support of this we furthermore found that Rest was required for PcG protein binding to a highly significant number of Rest binding sites in mES cells as well as for the binding to a selected number of neuronal target genes in NT2-D1 cells. Taken together our observations, strongly support a role for Rest as a DNA specific PcG recruitment factor.

To understand if PRC2 was required for Rest-mediated PRC1 binding, we took advantage of the *Eed−/−* mES cells, which lack PRC2 activity and therefore the H3K27Me3 mark [53]. Accordingly, PRC1, which is recruited to H3K27Me3 marked chromatin regions through the chromodomain-containing CBX proteins, was expected to be displaced from chromatin in cells lacking Eed. Nonetheless, when *Eed−/−* mES cells were compared to *Wt* mES cells, the Rnf2 signal strength was only marginally affected at Rest binding sites, although roughly 9 out of 10 Rnf2-binding sites were lost in *Eed−/−* mES cells (Figure 6A and 6B). This interesting finding demonstrated that Rest-dependent recruitment of PRC1 to chromatin occured independently of PRC2 activity and the H3K27Me3 mark, and offers a mechanism that, in part, could explain why *Wt* and *Eed−/−* mES cells have similar levels of the PRC1 catalyzed H2AUbi mark [19].

Given the aforesaid recruitment of PcGs to Rest binding sites and our biochemical data showing interaction between Rest, PRC2 and PRC1, we were highly puzzled by the observation that the absence of Rest, at a subpopulation of Rest binding sites, resulted in an increased binding of both PRC1 and PRC2 members in the *Rest−/−* mES cells. Interestingly, comparisons with matched control regions, revealed that both gain and loss of PcG binding in the *Rest−/−* mES cells were highly significant (except Suz12 loss) and specific to Rest binding sites. This suggested that the changes in PcG binding between *Wt* and *Rest−/−* mES cells indeed was a result of the absence of Rest and that Rest recruited PcG proteins to some genomic loci, while limiting the binding to others. In agreement with the view that PRC1 is recruited downstream of PRC2, the increase in Rnf2 binding at some Rest binding sites were linked to a similar increase in Suz12 binding at these sites (Figure S3). Moreover, when the influence of the position relative to TSS or CpG-islands was compared to that of PRC2 levels, it was clear that the gain in Rnf2 binding observed at Rest binding sites, close to TSS and CpG-islands, were indirect consequences of increased PRC2 recruitment to these entities (Figure 5E). Based on this, and the genome-wide analyses of Rnf2 in *Eed−/−* mES cells, we propose that PRC2 is dispensable for the majority of PRC1 recruitment far from CpG-islands, whereas PRC2 is the prime mechanism responsible for CpG-island proximal PRC1-recruitment, and only a minority of the CpG-island proximal Rnf2 binding sites is instead depending on alternative mechanisms, such as Rest. The observed gain of PRC2-signal at many Rest binding sites in the *Rest−/−* mES cells, suggest that Rest not only serves as a factor recruiting PcG complexes, but can directly or indirectly limit the amounts of PcG proteins on chromatin near some of its binding sites. Indeed, Rest is known to interact with other co-repressors (HDACs, CoRest/Lsd1 and G9a), which may affect chromatin structure in a way that limit PRC2 binding. It is tempting to speculate that this is part of a mechanism, which has evolved to increase robustness in gene regulation by compensating for the loss of one repressive complex, and thereby ensure redundancy and fidelity in the silencing of lineage specific genes. This might also be the reason for the relatively modest effects of *Rest* knock-out or knock-down on gene expression in mES cells [41] compared to the more pronounced changes observed in the NT2-D1 cells (Figure 2B). In line with this, using the expression analyses data from Færk et al. [41], looking at genes with either gain or loss of Rnf2 or Suz12 from our ChIP-seq analysis, we found no significant effects on gene expression correlating with the change in PcG binding in mES cells (Figure S6), although these genes are regulated by PcG according to Leeb et al. (Figure S7).

During the final preparation of our work a study from the Kerppola laboratory was published [54], which supports our findings of an interaction between PRC1 and Rest. Based on a selected number of genes, divided into either promoter proximal- or promoter distal-Rest binding sites, they concluded that Rest inhibits PRC1 recruitment at proximal binding sites, while recruiting PRC1 at distal binding sites. Since promoter proximal elements are rich in CpG islands this conclusion is in agreement with our genome-wide analysis of PcG protein binding and annotated CpG islands, which showed that Rest is required for PRC1 binding in the absence of CpG islands. However, it should be noted that whereas the Ren et al. study, on the basis of relatively few selected target genes, gives the impression that all Rest binding sites co-localize with PRC1, our genome wide analysis show direct overlap of Rest and PRC1 on 165 Rest binding sites out of a total of 3,378 Rest binding sites identified in our ChIP-sequencing analysis in mES *Wt* cells. Of these 165 co-localizing sites, 80 were found outside promoter proximal CpG islands and 85 on CpG islands. Furthermore, our study revealed that the increase of PRC1 (Rnf2) at promoter proximal Rest binding sites correlated with an increased recruitment of PRC2 to CpG islands at these locations. The increase in PRC2 was not directly translated into increased H3K27Me3 as one might expect, which suggest that H3K27Me3 in those regions were already quite high. Therefore, it is possible that the recruitment of PRC2 to these CpG rich regions is controlled by other histone modifications mediated by Rest complexes, which prevent efficient binding of PRC2 to the H3K27Me3 marked regions [55]. Furthermore, related to the fact that only about 5% of the Rest binding sites in mES cells showed co-localization with Rnf2, although PRC1 complexes are very abundant, suggest that other factors have influence on the recruitment of the Rest-PRC1 complex. Even though, we found that the Rest-PcG protein complexes were biochemically stable in the absence of ncRNAs (RNase treatment) it is still possible that ncRNAs such as *HOTAIR* could play a significant role in stabilizing Rest-PcG complexes on chromatin and thereby influence target gene specificity. Considering that Rest binding sites might be part of mammalian PRE elements, it is furthermore likely that other transcription factors, binding in the vicinity of Rest and with affinity for PcG complexes, influence whether or not PRC1 is stably interacting with REST on a particular binding site. Since our HAN11 double-tag affinity purification revealed a number of other transcription factors beside REST, future studies will aim at investigating if any of those can co-operate with REST in targeting PcG complexes to specific genomic loci.

In conclusion, we have shown that the transcription factor REST interacts with PRC1- and PRC2-complexes, interactions that we found to be independent of ncRNAs. Importantly, our data furthermore showed that the PRC1 complex can be recruited to a number of Rest binding sites independently of PRC2 activity and CpG islands. Surprisingly, we also found that there exist a CpG-island-associated increased recruitment of PRC2 in the absence of Rest, at a number of genes. We propose that this up-regulation of PRC2 binding functions to prevent unscheduled activation of differentiation specific genes and contribute to the robustness of mES cells. To understand the details regarding this compensatory mechanism and whether genomic regions, recruiting REST-PRC1 independently of PRC2 activity, are part of mammalian Polycomb Responsive Elements (PREs) will be the focus of future experiments.

## Materials and Methods

### Cell culture

Mouse embryonic stem (mES) cells were cultured on 0.1% (w/v) gelatin-coated plates in Glasgow medium (Sigma) supplemented with glutamax-1 (Gibco), non-essential amino acids (Gibco), 50 µM 2-mercaptoethanol–PBS, 15% ES-cell-qualified FBS (Gibco) in the presence of 1,000 U/ml of LIF (Millipore), and 1% (v/v) pen/strep. In addition, the *Rest*−/− and *Wt* control mES cells were cultured in the presence of feeder cells (Mitomycin C treated primary mouse embryonic fibroblasts). The mES *Eed*−/− and Rnf2−/− mES cells were provided by Dr. Anton Wutz. The *Rest*−/− and *Wt* control mES cells were provided by Dr. Zhou-Feng Chen and Dr. Helle Færk Jørgensen. HEK293FT and NT2-D1 cells were grown in DMEM (4.5 g/l D-glucose, Gibco) supplemented with 10% (v/v) FCS and 1% (v/v) pen/strep.

To induce neuronal differentiation of NT2-D1 cells, exponentially growing cells were seeded at 30% confluency and 24 hours later retinoic acid (RA) was added to a final concentration of 10 µM. Medium was changed every second day.

### Size-exclusion chromatography and immunoprecipitation

Cells or nuclear preparations were lysed in high-salt (HS) lysis buffer (50 mM Tris, pH 7.2, 300 mM NaCl, 0.5% (w/v) Igepal CA-630, Leupeptin (1 µg/ml), Aprotinin (1 µg/ml), 1 mM PMSF, 1 mM EDTA (pH 7.4)). After a short 2 second ultrasonication pulse (Branson Sonifier; 20% max amplitude) the lysates were left on ice for 30 min. After centrifugation at 20,000 g (4°C) for 15 min and 100,000 g for 30 min the lysates were passed through a 0.45 µm low protein binding filter (Ultrafree MC spinfilter, Millipore) followed by a 0.22 µm filter before the protein concentration was determined by Bradford assay (BioRad). Between 5 and 10 mg of protein was loaded on a Superose 6 HR 10/300 (24 ml) equilibrated in GF buffer (25 mM Tris, pH 7.2, 150 mM NaCl, 0.2% (w/v) Igepal CA-630, 1 mM EDTA (pH 8.0), 1% (w/v) glycerol) in 1 ml (flowrate 0.3 ml/min). One ml fractions were collected (4°C) and characterized by Western blotting and afterwards processed for immunoprecipitation by pooling peak fractions of interest. Typically 100–200 µl (pooled fractions) was used for each immunoprecipitation using anti-REST or control IgG. For direct Western blotting, to visualize the elution profile of individual proteins, an equal volume of each fraction was mixed with 2X LSB and heated at 95°C for 5 min. Ten µl was loaded per lane for SDS-PAGE and Western blotting. All SDS-PAGE gels used were precast 4–12% gradient gels or 10% homogenous gels (Invitrogen) using the MES buffer system.

Anti-REST and general IgG (rabbit; DAKO) were pre-coupled to proteinA-Sepharose beads (0.5 µg antibody per 40 µl 1∶1 slurry) and cross-linked using DMP (dimethyl pimelimidate dihydrochloride, Fluka) at pH 9.0 in 200 mM borate buffer according to standard procedures.

For samples treated with RNase V1 and RNase A the immunoprecipitates were washed twice in HS buffer followed by two washes in HS buffer adjusted to 500 mM NaCl and ones in HS buffer before adding 0.1 Unit of RNase V1 (Ambion) and 0.1 Unit of RNase A (Roche) in HS buffer. Samples were incubated at 10°C for 30 min followed by one wash in HS buffer, one wash in 500 mM HS buffer and one final wash in HS buffer. Samples were eluted in 2X LSB and heated to 95°C for 5 min before SDS-PAGE and immunoblotting.

### Quantification of mRNA levels by qPCR

RNA was purified using RNeasy Plus Mini kit (Qiagen) and cDNA was generated by RT–PCR. Quantifications were done using the Fast SYBR Green Master Mix (Applied Biosystems) and an ABI Step One Plus. *Beta-Actin* was used for normalization. The sequences of the primers used can be found in Table S8.

### ChIP assays

Cells were fixed for 10 min in culture media containing 1% formaldehyde and were processed for ChIP as previously described [44]. Antibodies used: Rabbit IgG (DAKO), rabbit anti-H3K27Me3 (Cell Signalling, C36B11), rabbit anti-H3 (GERA; antigen sequence: CGIQLARRIRGERA), rabbit anti-REST (Millipore, 07-579), rabbit anti-RNF2 (NAST; antigen sequence: NASTHSNQEAGPSNKRTKT), rabbit anti-SUZ12 (Cell signaling), rabbit anti-Jarid2 (Novus Biologicals), anti-CBX7 (“RELF”, [44]), anti-CBX8 (“LAST”, [44]), anti-NSPC1 (XW5).

### Western blotting

Western blotting was performed according to standard procedures using the following antibodies: Anti-RNF2 (“NAST”), anti-REST (Millipore, 07-579), anti-EZH2 (BD43-43), anti-OCT4 (ab19857, Abcam), anti-GAPDH (6G5, Biogenesis), anti-TUBULIN (Sigma, T6074), goat anti-SUZ12 (Santa Cruz, sc-46264), anti-BCOR (Novus Biologicals, NB100-87005), anti-CBX7 (“RELF”, [44]), anti-CBX8 (“LAST”, [44]), anti-NSPC1 (XW5), anti-E2F6 (TFE61), anti-BMI1 (DC9), anti-EED (AA19). Blots were developed using HRP-conjugated anti-rabbit, mouse or goat antibodies, depending on the species of the primary antibody, and enhanced chemiluminescence (ECL; Pierce). All exposures were done using Hyperfilm (Amersham).

### ShRNA and lenti virus production

VSV-G virus particles were generated by calcium phosphate-mediated co-transfection of PAX8 and VSV-G plasmids together with the pLKO.1 targeting construct in 293FT cells. pLKO.1 targeting constructs: pLKO.1-RNF2 (TRCN0000033697, Sigma), pLKO.1-REST (TRCN0000014783, Sigma) and pLKO.1-RCOR1 (TRCN0000147184). An empty pLKO.1 vector was used as control. NT2-D1 cells were incubated with virus supernatant for 8 hours and selection using 2 µg/ml puromycin started after 24 hrs PI. The NT2-D1 cells were harvested for ChIP, WB, and RNA after 4 days of selection.

### Affymetrix microarray

Total RNA was extracted from NT2-D1 cells treated with pLKO.1-Ct or shRNA to knockdown *REST* and *RNF2* mRNA. RNA was prepared from 3 independent experiments. 300 ng of total RNA from each experiments was processed for microarray expression analysis according to Affymetrix standard procedures. Up-regulated genes were identified using Microsoft Excel 2003 by individual probe-sets showing more than a two-fold change compared to control and with p-values below 0.05 in Student's t-test. Cluster analysis was performed using BRB-ArrayTools v3.81 (http://linus.nci.nih.gov/BRB-ArrayTools.html) using standard settings. Before cluster analysis probe sets were first filtered for 1) at least one array with a read above 10 and 2) more than one array deviating at least 1.5 fold from average.

### ChIP sequencing

DNA from three parallel ChIPs were pooled and 10 ng was used for making ChIP-seq libraries. Libraries were generated according to Illumina recommendations and sequencing was done on a Genome Analyzer II (Illumina). High quality reads (Chastity score > = 0.6) were aligned to the mouse genome (mm9) using Eland (Illumina) allowing up to two mismatches within the first 32 bases. Reads not aligning uniquely to the mouse genome were removed and profiles were presented using the UCSC Genome Browser (http://genome.ucsc.edu/) [56]. For detailed information about data handling and analyses see Procedure S1.

## Supporting Information

Figure S1 Biochemical analyses of HAN11 and REST-RNF2 complexes. (A) Double-tag purification of Flag-Ha tagged HAN11 expressed in HEK293 cells, using anti-Flag (M2) and anti-HA (12CA5) antibodies. 20% of the co-purified proteins were used for visualization by silver staining and 60% for Mass Spectrometry identification. (B) PRC1 members and transcription factors identified in the HAN11 double-tag purification. (C and D) Anti-Flag immunoprecipitations were performed on lysates from HEK293 cells expressing either Flag-Ha-REST (FH-REST) alone or in combination with GAL4-RNF2 or the three core members of the PRC2 complex (HA-EED, EZH2 and SUZ12). Immunoprecipitates were divided in halves and subsequently incubated in the presence or absence of RNase (C) or Ethidiumbromide (D). Samples were analysed by Western blotting using antibodies as indicated. (E) Nuclear extracts from HEK293 cells were processed for size-exclusion chromatography followed by Western blotting to reveal the profiles of Polycomb proteins and the transcription factors REST. Pooled fractions (1: Fractions F8–9; 2: F12–14; 3: F20–22) were used for immunoprecipitation (IP) with anti-REST or control IgG and processed for Western blotting with antibodies as indicated (total lysate: 12 µg of protein). (F) Nuclear extracts from HEK293 cells were processed for size-exclusion chromatography followed by Western blotting to reveal the profiles of RNF2 and BMI1 and the transcription factors REST. Pooled fractions (Fractions F7–8; F13–14) were used for immunoprecipitation (IP) with anti-REST, anti-RNF2 or control IgG and processed for Western blotting with antibodies as indicated (total lysate: 12 µg of protein).
(EPS)

Figure S2 ChIP analyses for PRC1. (A) Direct ChIP for the PRC1 members Cbx7, Cbx8 and Nspc1 in E14 *Wt* and *Eed* −/− mES cells. (B) Cbx7 ChIP-seq profiles from E14 *Wt* and *Eed*−/− mES cells. (C) Direct ChIP for Cbx7 and Cbx8 in *Wt* and *Rest−/−* mES cells.
(EPS)

Figure S3 Binding density analyses. (A) Color-coded density plots showing the difference in Rnf2 ChIP-seq signal in *Wt* and *Rest−/−* mES cells relative to Suz12 (*Wt*), Suz12 (*Rest−/−*), Jarid2 (*Wt*), Jarid2 (*Rest−/−*), K27me3 (*Wt*), and/or K27me3 (*Rest−/−*) signal at the 395 Rest peak positions that were scored Rnf2-positive. Densities depend on the number of peaks, whereas the color-coding corresponds to the average Rnf2*_Rest−/−_*/Rnf2*_Wt_*-ratio with red, green and blue illustrating increased, unaffected, or decreased signal, respectively. The signal intensity was quantified either within the Rest-peak position (caption = “REST-peak only”) or in a 2 Kb region centered around the Rest-peak (caption = “entire 2 Kb region”), and Peaks were binned in 1.5 fold bins depending on the two signals marked at the x- and y-axis. (B) Left panel: Excerpt from (A) showing the difference in Rnf2 ChIP-seq signal in *Wt* and *Rest−/−* mES cells relative to Suz12 (*Wt*) and K27me3 (*Wt*) average intensity in the 2 Kb region centered around the Rest-peak. Four frames termed A, B, C, and D mark the bins, which are used for the graphs in the right panel. Right panel: Graphs illustrating the average ratio between Rnf2 ChIP-seq signal in *Rest−/−* and *Wt* mES cells relative to either Suz12 (*Wt*) (A, B) or K27Me3 (*Wt*) (C, D) average intensity from peaks having similar levels of K27Me3 (*Wt*) (A, B) or Suz12 (*Wt*) (C, D). Numbers in blue refers to the number of peaks that are used for each data point. Note H3K27Me3 correlates poorly to the average Rnf2-ratio, whereas the average Rnf2-ratio is clearly correlated to the Suz12 level.
(EPS)

Figure S4 ChIP sequencing analyses. (A) ChIP-seq profiles for H3K27Me3, Rest, Rnf2, Suz12 and Jarid2 in *Wt* and *Rest*−/− mES cells for the examples shown in Figure 6. CpG islands are indicated in green color. (B) ChIP-seq profiles for Rest and Rnf2 in E14 *Wt* and *Eed−/−* mES cells for the examples shown in Figure 3. Positions of CpG islands are indicated (green color).
(EPS)

Figure S5 Gene Ontology (GO) analysis for genes with Rest and Rnf2 co-localization and/or Rest and Suz12 co-localization within 5,000 bp of Rest peaks in *Wt* mES cells.
(EPS)

Figure S6 Graphs illustrating average change in the expression of genes, bound by Rest in relation to Rnf2 (A) and Suz12 (B) loss or gain, as well as the position of the Rest binding sites relative to CpG islands. Left and right panels show the log2 fold change in gene expression in *Rest−/−* and Rest knockdown relative to *Wt* and control mES cells, respectively. Expression data are from [41]. Error bars illustrate standard error of the mean and p-values were calculated using Student's t-test.
(EPS)

Figure S7 Gene expression data from *Wt, Eed KO, Rnf2 KO and Double KO* mES cells (Leeb et al 2010). (A) Expression of *Best2*, *Brunol6*, *Prrxl1*, *Calb1*, *Nudt9* and *Stag3* genes in different genetic background: *Wt*, *Eed−/−*, *Rnf2−/−*, and [*Eed−/−*, Rnf2−/−] double KO mES cells. (B) Average gene expression for genes scored positive for Rnf2 and Rest. Rest-peaks that scored positive for Rnf2 (see Figure 4B), were subdivided into categories depending on the Rnf2-ratio (*Rest−/−*/*Wt*), and the average log2 changes in expression level were calculated from the Leeb et al., 2010 (probe-sets used correspond to the genes closest to the Rest-peak).
(EPS)

Procedure S1 Large-scale purification of FLAG-HA-HAN11. ChIP-sequencing data handling and analysis. Dataset normalization. Co-localization. Average distribution profiles. Heat-maps. Co-occurrence matrices. cRNA preparation and in vitro transcription. Array hybridization and scanning. Normalization (microarray).
(DOC)

Table S1 Expression profiling in NT2-D1. DNA microarray expression analysis for NT2-D1 cells treated with a control shRNA or a shRNA against *REST* or *RNF2*. Supporting material for Figure 1D.
(XLS)

Table S2 Rest peaks and co-localization with Rnf2, Suz12 and Jarid2 in *Wt* mES cells. Supporting material for Figure 3 and Figure 4.
(XLS)

Table S3 Ratio measurements of PcG ChIP-seq signals in *Wt* versus *Rest*−/− mES cells. Supporting material for Figure 4B.
(XLS)

Table S4 Rest and Rnf2 co-localization >1 Kb or <1 Kb from CpG islands. Supporting material for Figure 4C.
(XLS)

Table S5 Rest peaks in E14 *Wt* mES cells. Supporting material for Figure 5.
(XLS)

Table S6 Lists of all Rnf2 peaks identified in E14 *Wt* and *Eed*−/− mES cells. Supporting material for Figure 5.
(XLS)

Table S7 Gene Ontology (GO) analysis for genes with Rest and Rnf2 co-localization and/or Rest and Suz12 co-localization in the *Wt* mES cells cultured with feeder cells. Co-localization within 5,000 bp of the Rest peak. Supporting material for Figure S2.
(XLS)

Table S8 List of primers used for real time quantitative PCR. Supporting material for the Materials and Methods section.
(XLS)

Table S9 Gene Ontology (GO) analysis for genes co-regulated by REST and RNF2 in NT2-D1 cells.
(XLS)
