# Supplementary material for: REST–Mediated Recruitment of Polycomb Repressor Complexes in Mammalian Cells
Source: PLoS Genet. 2012 Mar 1;8(3):e1002494. doi: 10.1371/journal.pgen.1002494 (PMC3291536; doi:10.1371/journal.pgen.1002494)
Supplement: Procedure S1 — Large-scale purification of FLAG-HA-HAN11. ChIP-sequencing data handling and analysis. Dataset normalization. Co-localization. Average distribution profiles. Heat-maps. Co-occurrence matrices. cRNA preparation and in vitro transcription. Array hybridization and scanning. Normalization (microarray). (DOC) [file pgen.1002494.s008.doc]

**Supporting Procedures S1**

Large-scale purification of FLAG-HA-HAN11.

293-T cells were transfected with pMinkio-FLAG-HA-HAN11 and cultured in the presence of 1 μg/ml puromycin. Nuclear pellets isolated from a puromycin selected culture were lysed in high-salt (HS-) lysis buffer (50 mM Tris, pH 7.2, 300 mM NaCl, 0.5% Igepal Ca-630, 1 mM DTT, 5 μg/ml Leupeptin, 5 μg/ml Aprotinin, 1 mM PMSF) at 4°C and briefly sonicated (20% max amplitude for 2 sec in a Branson ultrasonicator). After centrifugation (20,000 g for 15 min and 100,000 g for 1 hour) the lysate was precleared by incubation (end-over-end) with mouse IgG cross-linked to protein G-Sepharose for 2 hours. After removing the beads by a short pulse-spin, the supernatant was spun at 20,000g for 15 min followed by incubation with anti-Flag antibodies (M2 agarose beads, Sigma) at 4°C overnight. Following extensive washing in HS-lysis buffer Flag-HA tagged HAN11 was specifically eluted using 2 mg/ml Flag peptide in HS-lysis buffer (2 hours at 10°C). The eluate was then incubated with mouse IgG protein G-Sepharose beads for 3 hours, followed by anti-HA (12CA5) protein G-Sepharose beads overnight (4°C). After washing in HS-lysis buffer the mouse IgG beads and the anti-HA beads (Sigma) were eluted with 2 mg/ml HA peptide in 50 mM NH_4_HCO_3_ for 4 hours at 4°C. The eluted material was freeze-dried and dissolved in Laemmli sample buffer, boiled and separated by SDS-PAGE, gel was silverstained and sample was processed for mass spectrometry analysis.

**Affymetrix microarray**

cRNA preparation and in vitro transcription.

300 ng total RNA was used as starting material for the cDNA preparation. The first and second strand cDNA synthesis was performed using the 3´ IVT express Kit (Affymetrix) according to the manufacturers instructions. Labelled cRNA was prepared using the 3´ IVT express Kit (Affymetrix) according to the manufacturers instructions.

Array hybridisation and scanning.

15 µg of cRNA was fragmented at 94^0^C for 35 min in a fragmentation buffer containing 40 mM Tris-acetate pH 8.1, 100 mM KOAc, 30 mM MgOAc. Prior to hybridisation, the fragmented cRNA in a 6xSSPE-T hybridisation buffer (1 M NaCl, 10 mM Tris pH 7.6, 0.005% Triton), was heated to 95^0^C for 5 min and subsequently to 45^0^C for 5 min before loading onto the Affymetrix HG_U133 2.0 probe array cartridge. The probe array was then incubated for 16 h at 45^0^C at constant rotation (60 rpm). The washing and staining procedure was performed in the Affymetrix Fluidics Station 450. The probe array was exposed to 10 washes in 6xSSPE-T at 25^0^C followed by 4 washes in 0.5xSSPE-T at 50^0^C. The biotinylated cRNA was stained with a streptavidin-phycoerythrin conjugate, final concentration 2 mg/ml (Molecular Probes, Eugene, OR) in 6xSSPE-T for 30 min at 25^0^C followed by 10 washes in 6xSSPE-T at 25^0^C An antibody amplification step followed using normal goat IgG as blocking reagent, final concentration 0.1 mg/ml (Sigma) and biotinylated anti-streptavidin antibody (goat), final concentration 3 mg/ml (Vector Laboratories). This was followed by a staining step with a streptavidin-phycoerythrin conjugate, final concentration 2 mg/ml (Molecular Probes, Eugene, OR) in 6xSSPE-T for 30 min at 25^0^C and 10 washes in 6xSSPE-T at 25^0^C. The probe arrays were scanned at 560 nm using a confocal laser-scanning microscope (Affymetrix Scanner 3000). The readings from the quantitative scanning were analysed by the Affymetrix Gene Expression Analysis Software.

Normalization.

The readings from the quantitative scanning were RMA normalized using the Affymetrix Expression Console v1.1.

ChIP-sequencing data handling and analysis.

Dataset conversion and filtering for multiple reads was done in Microsoft Excel 2003 using custom made Visual Basic macros. Where more than one read mapped to the same position and at the same strand duplicates were removed. Peaks were identified using CisGenome v1.2 software {Ji, 2008 #102} according to the guidelines for two-sample peak-finding at the authors website (http://www.biostat.jhsph.edu/~hji/cisgenome/index.htm), with inclusion of IgG control ChIP-sequences from the same chromatin preparation as a negative control, in order to improve detection quality and reduce unspecific peaks. Dp0Hat-values and thresholds were established in a prior two-sample dataset exploration using CisGenome, and 100 bp windows, 25 bp step-sizes and a FDR values of 0.1 was used as standard. For the data from *Rest* -/- and corresponding *Wt* control mES cells, we had to deviate from this scheme because the datasets were too big for exploration and peak-calling. Therefore the size of the datasets were paired, so that the largest of each antibody dataset was reduced to the size of the smallest. Subsequently, the reads of each dataset were divided into two parts based on chromosome number, and peaks were called as above but using a fixed threshold (15 reads). After peak-calling, all peak-sets were cleared of unspecific regions, by colocalizing them to IgG peak-sets from *Wt* and *Rest* -/- samples (as described below), and peaks that were within 10,000 bp of an IgG were excluded from further analysis.

Dataset normalization.

For quantitative analyses comparing several dataset, data was normalized to the total number of non-duplicate reads of the dataset multiplied with 200bp and divided by the size of the mouse mm9 genome, which was set to 2,716,965,481 bp according to UCSC Genome Bioinformatics (http://genome.ucsc.edu/).

Co-localization.

Co-localization and gene annotation to the mouse mm9 genome was done in Excel 2003 using custom made Visual Basic macros, and Refseq and CpG track data were derived from the Genome Browser at UCSC (http://genome.ucsc.edu/). For each peak in a particular data set the closest gene as well as peak in the other data set was identified. Distances between peaks were calculated from the nearest peak-border.

Average distribution profiles.

Average profiles over Rest peaks were done in Excel 2003 using custom made Visual Basic macros. First the coverage of each dataset was calculated by setting the average DNA fragment length to 200 bp and extending the read accordingly. Secondly the coverage was sampled from -3,000 bp to +3,000 bp of the center of each peak with 25 bp resolution or at from -1,000 bp to +1,000 bp of the center of each peak with 10 bp resolution, with positive values representing the direction towards the TSS of the nearest gene. Average reads at each position was calculated, and where mentioned, the reads were normalized to the average read in the IgG control samples.

Heat-maps.

Heat-maps for individual profiles, were made as described above for average profiles. Images of the tracks were generated using a ImageJ (Rasband, W.S., ImageJ, U. S. National Institutes of Health, Bethesda, Maryland, USA, http://rsb.info.nih.gov/ij/, 1997-2009.) and a custom-made macro. The intensity of the images were pairwise (for each antibody) normalized to the size of the datasets, and the ratio calculated and pseudocoloured using custom-made ImageJ macros.

Co-occurrence matrices.

The maps are based on scans with a 2500 bp window along different positions near the transcription start sites (TSS) or transcription end points (TE) annotated in the mouse genome (mm9). For each scan the number of observed genes positive (1 or more peaks within the window) for both Rest and Rnf2 were calculated and normalized to the number expected by chance based on the frequencies of genes positive for Rest and Rnf2 alone in that particular position relative to the TSS or TE. Scans with a higher or lower frequency than expected from random localization were coloured blue or red, respectively. Scans with a P-value below 0.05 (Bonferroni corrected for multiple sampling), are overlaid with transparent green. Images of the co-occurrence matrices were generated using a ImageJ and a custom-made macro.
